# The Relation of the General Practitioner to the Specialist in Dentistry

**Published:** 1921-01

**Authors:** A. W. Thornton


### THE RELATION OF THE GENERAL PRACTITIONER TO THE SPECIALIST IN DENTISTRY

BY A. W. THORNTON, D.D.S.

At the present time the whole field of practice has been divided and subdivided until it is almost impossible to differentiate between the many specialists of the present day. It is true that with the increase of knowledge this division became necessary, but division and subdivision have seemingly run riot during the present age. There are reasons for this condition. There is an unwillingness on the part of men to pay the price for knowledge. To get a wide knowledge means that a man must burn the midnight oil; so men are moving in the line of least resistance. Instead of trying to cover a wide field and become generally proficient, they narrow the field of endeavor and hope to cover it. In that way specialists have grown up. Then there is the advanced standard of living. Men and women today are willing to pay the price for the best goods and the best class of service; men realize that if they call themselves specialists and live up to their calling, they can obtain increased fees for their services, and that has had a great deal to do with the subdivision of medicine and dentistry into various classes or specialties.

As to the specialties in dentistry, there is the orthodontist; then we have the pyorrhea specialist; the crown and bridge specialist; then the exodontist; then the prosthodontist; and recently the men who are engaged in pyorrhea work have called themselves periodontists. In fact, there are so many ''dontists'' that it is only with a good deal of courage, and almost an expression of regret, that some of us call ourselves dentists.

In my estimation—and I want to discuss this matter frankly with you—many of these subdivisions are absolutely unnecessary. As I see it, there is not room for more than two specialties in dentistry, one the specialty of orthodontia, and the other the specialty of oral surgery. Now this involves no reflection on the men who feel that they have a special aptitude for some particular calling. But it does mean that outside of these two specialties the profession of dentistry is sufficiently limited to enable any man of ordinary intelligence and common industry to give satisfactory service in these various lines, with the exception of the two that I have just mentioned. Why do I except orthodontia and oral surgery? As to orthodontia, the reason is—of course, this is only my own opinion—that the practice of this specialty requires a special fondness for and ability to get along with children. Every man does not possess that fondness and that ability.

Now, the orthodontist carries on his work largely with children, and he must be a man specially qualified to deal with them. Moreover, the practice of the specialty of orthodontia calls for, perhaps, a closer special study than any of the other mechanical branches of dentistry. It calls for a high standard of mechanical ability, a very accurate knowledge of the anatomy of the parts affected, and an absolute honesty of purpose in dealing with the patients and the parents of the patients. The work of the orthodontist is not finished, as in the case of our ordinary practice, in a day or a week, or two or three weeks at the outside; he must carry on the work for years; therefore, it requires a man of peculiar temperament and peculiar ability to practice orthodontia. Then there is another reason: up to the present, at least, I do not think that the work carried on in our schools has been sufficiently broad to enable a man to go out and practice orthodontia as successfully as he could prosthetic or operative dentistry.

In connection with this, as in connection with every other specialty, I may say that no man should go out from college and enter upon the practice of a speciality until he has had at least some years' experience in general practice. Now, why? Well, for this reason: the tendency of all specialists is to become lop-sided—that is unavoidable. "As a man thinketh in his heart, so is he;" if he thinks orthodontia, then to him orthodontia is the biggest thing in life. If he thinks pyorrhea, then to him pyorrhea is the biggest thing in life. Then there is the inability of the specialist who has entered upon the practice of his specialty immediately after graduating to see the conditions from the standpoint of the general practitioner. Therefore, in order that he may have the confidence of the men who will send him their patients, it is necessary that he should have some years at least in general practice.

Now, for the case of the oral surgeon—that is, the man who will be able to operate for cleft palate, for hair lip, and for all the deformities that may occur in and about the mouth. Just there we approach the border line between dentistry and medicine, as this latter term is usually used. The question arises: Is oral surgery a specialty in medicine or a specialty in dentistry? I have had a number of requests recently from all over the country inquiring whether dentists could come to Montreal and get some special work in oral surgery. Now, that is not possible for a man who is not trained in medicine and in surgery; a knowledge of both, it seems to me, is an absolute necessity. The essential thing in these operations is the condition after treatment and the possibilities of restoration. This demands the services of men fully trained in both sciences, but with their chief interest centered in dentistry. In the final analysis the permanent condition must rest with the skilled dentist. Now, in order to be an efficient specialist in that line, a man must be a general surgeon as well as a special surgeon. You could not imagine a man calling himself a general surgeon if he operated, say, only in cases of appendectomy. You could not imagine a man calling himself a specialist who would only operate for hernia or for hemorrhoids. These men are skilled surgeons, so we must have a man who is versed in the whole field of surgery, and who can make a success of his work if he goes into oral surgery, as Dr. Brophy, Dr. Gilmour, and other men are doing. I hope that in the near future we shall have in all our large cities, such as Ottawa, Montreal, Toronto, and so on, men who are trained and licensed both in medicine and in dentistry, and who will be giving their chief attention to mouth conditions, carrying along their intensive work in the line of dentistry.

Now we come to the question of the periodontist. In my opinion the work of the periodontist should never be divorced from that of the ordinary practitioner of dentistry; I believe that every man should do his own periodonto-clasia or pyorrhea work. This does not imply that I think that as good results are being obtained by the men in general practice as by those who are making a specialty of this work. I know that they are not. But it does imply that the man in general practice should be able to give just as good and intelligent service as the specialist, if he is willing to pay the price. On what do I base this assertion? There are many cases where a man is a general practitioner today and a specialist tomorrow. It is quite impossible that all who call themselves specialists should have assimilated a vast store of wisdom or manipulative ability, sufficient to cause a wide difference between the so-called specialists and the man in general practice. On the other hand, I do know that many of these specialists who feel a fondness for and a special aptitude in certain lines of work have given months or years of particular attention to certain classes of work before finally making up their minds to adopt this work as a specialty. There is a reason for this. One reason is the necessity for study, and work, and practice, to develop a high standard of fitness. Another reason is the necessity for obtaining a livelihood while preparing for the transition from general practice to the work of a specialist.

It is no longer possible to treat a case of pyorrhea, or interstitial gingivitis, or Riggs disease, or trench mouth, or Vincent's angina, or ulcerated stomatitis, or any of these conditions, by whatever name known, with the instruments usually found in the office equipment of the man in general practice, or in the time generally given to such cases. In altogether too many cases, such service is still too closely linked with the old idea of "cleaning a set of teeth" and with the old fee of fifty cents or a dollar.

There is another reason why the specialist is able to command a respect and a fee out of all proportion to that enjoyed by the men in general practice. Go to the office of any of the men in the various specialties, and then go into the majority of offices of the men in general practice, and you will discover for yourself many of the reasons for this difference of respect and appreciation. Our men in general practice must lay seriously to heart the conditions of the office, their nearness to approach to aseptic conditions in all their operations.

One of the best known dentists in this province, a man known to every one of you said to me a good many years ago: "I should not have been a dentist. I have not one single, solitary mechanical idea in my mind. I do the thing that I have been taught to do until I become fairly proficient in that thing. I continue doing it the same way until someone tells me of a better way; then I try that better way. But I have never been able to offer anything original myself.'' Well, that man is one of the most successful dentists we have—why? Because of his suavity of manner, his courtesy, his culture of mind, and because of the way he has always kept his office in the city in which he practices. As I have intimated, he is, perhaps, one of the most successful practitioners in the province at the present time.

There is a great need at the present time for a revival in study, so that the men who have been out of college for some time may have a chance to come in contact with recent discoveries in histology, pathology and bacteriology, and with the variations in treatment made necessary by these discoveries.

I do not know whether you had here within the last two years, or perhaps within the last year, a man who was giving instruction in nerve-blocking and in conduction anaesthesia. I do not know whether Dr. Smith has been here or not. I took his course in Toronto—not that I hoped to use it, but simply to keep in touch with the work and for the purpose of teaching students in the hospital. Then I took his course when he came to Montreal. Now, why did Dr. Smith meet with the success which attended his work everywhere he went? Simply because he was breaking virgin soil. The men whom he was teaching had practically no working knowledge of the anatomy of the head and face and neck. They had no idea of the area that would be anaesthetized by the injection, say, into the infraorbital foramen, into the mental foramen, into the anterior and posterior palatine, into the mandibular foramen; so the great part of his work was simply that of teaching the minute anatomy of these parts upon which they were working. So much have we laid that to heart that in McGill at the present time we are laying emphasis on that part of our teaching of anatomy. I think it is very important that a man should have a knowledge of the brain centers from which the nerves rise, their position in the brain, the parts of the anatomy supplied by the brain, the part that will be anaesthetized by making an injection into any one of these nerves, the depth of these foramina from the surfaces, the length of needle necessary, the part that will be anaesthetized.

I received a circular this week from a man who proposes to give a post-graduate course in the surgical extraction of teeth. Now, I think something of that kind is due at this time. Most of us have been extracting teeth in a manner which is anything but surgical. I think that a great advance is due, and can be brought about in this line. This man is giving a post-graduate course of four days for the modest sum of $200. Now, that looks like a good big fee. We gave Dr. Smith $1,800 for a week's work in Montreal. That looks like a good deal to pay for a week's work, but he was months and months down in Alabama, where there is no anatomy law, and where he could sit at the bedside until the patient died, call for the body two minutes afterwards, and prepare his specimens. He spent months down there doing work for which he did not get anything. So that if these men do qualify to give that kind of service which will enable you to go out and do better, why, we can afford to pay them a fairly good fee for the service they give.

This need of study is being supplied in many places by the study classes organized by the dentists in many American and Canadian cities. The method of conducting these splendid classes is as follows: a number of men form a class to study some phase of the regular work. After the fullest investigation these men divide the work into various steps, each member of the class becoming responsible for only one step in the operation, and doing this step so frequently that he becomes expert in it, and learns all its advantages and disadvantages, its easy parts and its difficult parts. So classes have been formed in prosthetic restoration, in the technique of inlay work, in conductive and block anaesthesia, in the treatment of pyorrhea, in removable bridgework, in partial denture work. Recently Dr. Nesbit, of Boston, gave a course of instruction in cast clasp work, setting forth what I believe to be so far the most suitable way of making restorations that the profession has yet learned of, getting away from the necessity of grinding down and mutilating teeth in order to put on shell crowns.

In order to keep abreast of the times it is quite necessary that many of our dentists should obtain a new perspective of their relation to their patients, to themselves, and to the profession. It is simply impossible for any man to keep abreast of the times without stated periods for study and observation. It is just as necessary, if a man is to attain to his own full stature, that he should spend a certain amount of money every year in study, in travel, in coming in contact with his fellow practitioners, in order to keep in touch with the newer developments, as it is that he should spend money for coal, or groceries, or clothing, or material, or for instruments for his office. Indeed, his ability to buy the things usually spoken of as the necessities of life will probably be in direct ratio to the time which he spends in study and improvement, because the latter expenditure will immeasurably increase his earning ability.

Specialists are here to stay. Some men are criticising them. Some who are suffering financially are finding fault. Most thoughtful men in the profession recognize the fact that a further sub-division would be a calamity, and that even some of those already in existence should revert to the general practitioner, whence they sprung.

In the final analysis, dental service is on a par with the bulk of commercial commodities. It is a question, not only of supply and demand, but a question of the excellence of the thing purchased. The whole world knows to-day the value of mouth hygiene, as well as the value and importance of a well-kept set of natural teeth. Because of this knowledge, those who can pay for dental service are going to have that service from the man who can render service of the highest standard, and that, in many cases, regardless of the fee that may be charged.

The important thing for each one of us to consider is this: am I capable of rendering to my patients as good service in the field in which many men are now calling themselves specialists as are the so-called specialists, or, if I am to be honest with these patients, must I send them to some other man, conscious that they will receive a standard of service which I can not personally give to them? The whole question resolves itself into two points—personality and efficiency.—Extracted from *Oral Health.*

